# Association between the executive dysfunction and balance function in patients with stroke

**DOI:** 10.1002/brb3.3542

**Published:** 2024-05-23

**Authors:** Katsuya Sakai, Yuichiro Hosoi, Yusuke Harada, Yuichi Kato

**Affiliations:** ^1^ Department of Physical Therapy, Faculty of Health Sciences Tokyo Metropolitan University Tokyo Japan; ^2^ Department of Rehabilitation Medicine Keio University School of Medicine Tokyo Japan; ^3^ Department of Sports Health Sciences Ritsumeikan University Kyoto Japan; ^4^ Department of Rehabilitation Reiwa Rehabilitation Hospital Chiba Japan; ^5^ Graduate School of Human Health Sciences Tokyo Metropolitan University Tokyo Japan; ^6^ Department of Rehabilitation Moriyama Neurological Center Hospital Tokyo Japan

**Keywords:** balance, executive dysfunction, mediation analysis, stroke patients

## Abstract

**Introduction:**

A previous study has shown an association between executive dysfunction (ED) and balance function in patients with stroke. However, it is unclear what factors mediate the association between ED and balance function. Therefore, the aim of this study was to investigate the association between ED and balance function and to identify mediating factors using mediation analysis.

**Methods:**

This study had a cross‐sectional design. The study included 107 patients with stroke. This study was divided into two groups (ED and non‐ED) using trail making test (TMT) part B. Two groups were compared for balance function (timed up and go test [TUGT] and Berg balance scale [BBS]) and other variables using paired test. In addition, partial correlation analysis with age, cognitive function as a control factor, and mediation analysis were also performed.

**Results:**

The ED group (N = 55) had significantly lower TUGT and BBS scores than the non‐ED group (N = 52). TMT part B correlated with TUGT (*ρ* = 0.41), BBS (*ρ* = −0.33), and Brunnstrom recovery stage (BRS) lower limb (*ρ* = −0.22). The TUGT model of mediation analysis showed a significant indirect effect as a result of mediation of the BRS lower limb between TMT part B and TUGT. The BBS model showed a significant indirect effect as a result of mediation of the activities of daily living (ADL) motor function between TMT part B and BBS.

**Conclusions:**

ED and balance function were associated, and the degree of paralysis and ADL motor function were associated with them in patients with stroke.

## Graphical abstract

Results of the model of mediation analysis. For the TUGT model, The mediation analysis showed that the correlation coefficient between TMT part B and TUGT decreased, indicating a significant BRS lower‐mediated effect. For the BBS model, the mediation analysis showed that the correlation coefficient between TMT part B and BBS decreased, indicating a significant FIM motor score‐mediated effect.

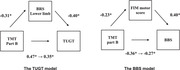

## 1 INTRODUCTION

Executive function is a higher brain function that involves several factors, including decision‐making, risk‐taking, planning, inhibitory control, working memory, and cognitive flexibility (speed, error processing, and attention) (Skidmore et al., 2023). The brain regions that control these include the prefrontal cortex, basal ganglia, and cerebellum, which are connected by cerebral white matter fibers (Friedman & Robbins, 2022; Povroznik et al., 2018). It has been reported that executive function is impaired in the elderly due to age‐related structural changes and reduced brain functional connectivity (Fjell et al., 2017). In the case of stroke, these areas are directly or indirectly damaged, resulting in executive dysfunction (Povroznik et al., 2018). These results in executive dysfunction, with impaired decision‐making, planning, inhibition, and cognitive flexibility. In particular, frontal lobe lesions are more likely to have more severe executive dysfunction than other lesions (Han et al., 2020). Han et al. (2020) investigated whether there is a difference in the degree of executive function between frontal lobe lesions and other lesions. They found that patients with frontal lobe lesions had poorer executive function than patients with stroke. These brain regions are affected, resulting in executive dysfunction in 25%–75% of stroke patients (S. Hayes et al., 2016; Skidmore et al., 2023). It has been reported that executive dysfunction leads to decreased ability to perform activities of daily living (ADL) and limits return to society (Ghaffari et al., 2021; Ownsworth & Shum, 2008). This is because executive dysfunction has been reported to affect walking and balance functions in patients with stroke.

Previous studies have shown an association between executive dysfunction and balance and walking functions in patients with stroke (S. Hayes et al., 2013, 2016). S. Hayes et al. (2013) and Sakai et al. (2023) investigated whether executive dysfunction was associated with the 10‐m walking time and dual‐task walking time. The results reported that executive dysfunction was associated with them. In addition, Sakai et al. (2023) investigated whether the 10‐m walking time differs according to the degree of executive dysfunction in patients with stroke. They found that the degree of executive dysfunction was associated with 10‐m walking time, with more severe executive dysfunction leading to longer walk times in patients with stroke. Thus, executive dysfunction was associated with walking ability in patients with stroke. Furthermore, executive dysfunction has been shown to be associated with balance function in patients with stroke (S. Hayes et al., 2016; Liu‐Ambrose et al., 2007). Liu‐Ambrose et al. (2007) and Sakai et al. (2023) examined executive dysfunction and determined balance function in community‐dwelling older adults after a mild stroke. They reported that executive dysfunction was associated with balance function. Hayes et al. (2016) used the behavioral assessment of the dysexecutive syndrome (BADS) to examine executive dysfunction and balance function (Berg balance scale [BBS]) in patients with stroke and found that executive dysfunction was associated with lower balance function. In addition, Sakai et al. (2023) used cluster analysis to classify executive dysfunction into three categories—mild, moderate, and severe—and investigated whether there were differences in balance function, as measured by the timed up and go test (TUGT), according to the degree of executive dysfunction in patients with stroke. They reported that balance function differed according to the degree of executive dysfunction. Thus, executive dysfunction was associated with walking and balance functions in patients with stroke. However, it is unclear through what factors executive function is related to balance function as measured by BBS and TUGT.

Mediation analysis is a method to investigate what factors are related between the dependent and explanatory variables (A. F. Hayes & Rockwood, 2017). Mediation analysis creates a model from the research hypotheses and allows one‐direction investigation of the involvement of the mediating variable between the dependent and explanatory variables (A. F. Hayes & Rockwood, 2017). Joyce et al. (2022) used mediation analysis to investigate whether psychological factors mediate and influence the effectiveness of rehabilitation in patients with chronic low back pain. The results revealed that perceived stress was effective in rehabilitation when it was a mediating factor. Thus, mediation analysis has the advantage of creating a model and clarifying causal relationships, and it can be used for disorder structure analysis and determining the effectiveness of rehabilitation. For the current study, factors related to executive function and movement and balance function have been reported to be the degree of paralysis and ADL function in patients with stroke (Lipskaya‐Velikovsky et al., 2018; Ownsworth & Shum, 2008; Sakai et al., 2023; Sánchez‐Herrera‐Baeza et al., 2023; Tanabe et al., 2022). Therefore, we hypothesized and modeled that these factors may be related to executive function and BBS or TUGT as mediating factors. By identifying mediating factors through this study, the causal relationship between executive dysfunction and balance can be clarified, which will help in assessment and planning intervention methods. Therefore, the aim of this study was to investigate the association between executive dysfunction and balance function in patients with stroke and to identify mediating factors using mediation analysis.

## 2 MATERIALS AND METHODS

### 2.1 Participants

This study had a cross‐sectional design. The participants were 135 patients with subacute stroke (mean age: 68.9 ± 12.5 years; males, 84; females, 51; time since stroke: mean, 72.5 ± 53.2 days). The study was conducted in two rehabilitation hospital units between August 2022 and December 2023. The sample size was calculated with a power = 0.80, *α* = 0.05, and an effect size = 0.390 (S. Hayes et al., 2016) using G power 3.1.9.2, which indicates that the ideal sample size should be larger than 46 participants. The inclusion criteria were as follows: (1) first‐time stroke; (2) age > 18 years; (3) presence of hemiplegia; and (4) no orthopedic disease. The exclusion criteria were as follows: (1) diagnosis of severe dementia and Alzheimer's disease; (2) diagnosis of higher brain dysfunction (e.g., unilateral spatial neglect, aphasia, and apraxia); and (3) age < 95 years. Participants received an explanation of the purpose of the study, and written informed consent was obtained before the study began. This study was approved by the ethics committee of Reiwa Rehabilitation Hospital and Moriyama Neurological Center Hospital (approval number: 00–10, 23002, approval date: July 25, 2022, and April 11, 2023, respectively), publicly registered in the UMIN Clinical Trials Registry (UMIN‐CTR) (trial registration ID: UMIN000048587), and adhered to the ethical standards established in the 1964 Declaration of Helsinki.

### 2.2 Executive function and cognitive assessments

Executive and cognitive functions were assessed using TMT parts A and B, the Mini‐Mental State Examination (MMSE), and the cognitive part of the functional independence measure (FIM).

TMT part A reflects motor speed and attentional function, while TMT part B has often been used to assess executive function (Reitan & Wolfson, 1985; Tamez et al., 2011). In TMT part A, the participants connected the circled numbers (1–25) in order as quickly as possible (Reitan & Wolfson, 1985; Tamez et al., 2011). In TMT part B, participants connected numbers and letters alternately in sequence as fast as possible (Reitan & Wolfson, 1985; Tamez et al., 2011). In this study, TMT part B was used as an indicator of executive dysfunction (Sakai et al., 2023, 2024).

The MMSE was used to assess cognitive function, with total scores ≥30 points indicating better cognitive function (Perneczky et al., 2006). MMSE scores of 0–10, 11–20, 21–23 or higher, and ≥24 indicated severe cognitive impairment, moderate cognitive impairment, mild cognitive impairment, and normal cognition, respectively (Perneczky et al., 2006).

FIM is an assessment tool that measures the ability to perform ADLs (Granger, 1986) and consists of FIM cognitive (5 items) and FIM motor items (13 items) (Granger, 1986). Cognitive items include memory, social problem solving, and communication and can assess social cognitive skills needed in daily life. Each item was scored on a six‐point scale (score range: 1–7), with 35, 91, and 126 being the highest scores for FIM cognitive, FIM motor, and FIM total, respectively. Higher scores indicate better functioning in ADL function.

### 2.3 Balance and other physical functions assessments

Balance functions were assessed using TUGT and BBS. Other physical functions were assessed using the Brunnstrom recovery stage (BRS) and the sensory part of stroke impairment assessment set (SIAS).

TUGT is an assessment tool for dynamic balance and walking functions (Hafsteinsdóttir et al., 2014). The participant was seated in a 40‐cm chair. The participant was instructed to stand up from the chair, walk to a cone 3 m away from the chair, walk around it toward their nonparalytic side, return to the chair, and sit down (Hafsteinsdóttir et al., 2014). The assessor instructed the participant to stand up from the chair at the “ready to go” cue and measured the time with a stopwatch. Participants were allowed to use walking aids and orthotics that they normally use in their daily lives. The TUGT was performed twice at maximum speed. The average TUGT was then calculated.

The BBS can measure the ability to maintain balance function (Berg et al., 1995). It comprises 14 items, and each item is scored on a five‐point scale (0–4), with a total score of 56 points. The BBS consists of basic movements such as standing up, one‐leg standing, and turning. Higher scores indicate better balance function.

The BRS assesses the degree of motor paralysis of the upper limb, finger, and lower limb functions (Brunnstrom, 1966) and consists of six items. The BRS had higher scores on this index (score range: 1–6), indicating better motor function.

The SIAS is a comprehensive tool for assessing motor and sensory function in stroke patients (Liu et al., 2002). The SIAS, which consists of 22 items, classifies functional disorders into nine types. In this study, only sensory items (tactile and position sensory) were used. This test was scored on a four‐point scale (score range: 0–3). The better the sensory function, the higher the score.

### 2.4 Date and statistical analysis

For TMT part B, two standard deviations (SD) were calculated. Participants were then divided into two groups: the executive dysfunction (ED) group, over 2 SD of the healthy participants score, and the nonexecutive dysfunction (NED) group, within 2 SD of the healthy participants score (Ashendorf et al., 2008). In addition, the number of groups was divided by the population number to calculate the prevalence.

The Shapiro–Wilk test was used to determine the data distribution. The Mann–Whitney *U*‐test was used to determine between‐group differences for TMT part A, B, TUGT, BBS, and various variables. The chi‐squared test was performed to assess categorical variables, including stroke type, paretic side, and sex.

Partial correlation analysis with age, MMSE, and FIM cognitive part as a control factor was performed to determine the relationships between TMT part B and TUGT and BBS and the other variables (BRS, SIAS, and FIM motor).

To identify factors mediating executive function in TUGT and BBS, two models were created, and mediation analysis was performed for each. The TUGT model has TUGT as the dependent variable, TMT part B as the independent variable, and BRS lower limb as the mediating factor. This is because executive function influences paralysis recovery and TUGT is associated with paralysis (Chan et al., 2017). The BBS model uses BBS as the dependent variable, TMT part B as the independent variable, and FIM motor as the mediating factor. The BBS model was used because executive function influences independence in ADL, and the BBS is associated with ADL function (E. H. Kim & Cho, 2023; Shao et al., 2024). The 95% confidence interval (CI) of the bootstrap method was used to test whether the mediating factor has a significant indirect effect on the dependent variable (2000 samples generated using nonparametric method) (A. F. Hayes & Rockwood, 2017).

Statistical analysis was performed using SPSS (version 29.0; SPSS Inc.) and mediation analysis using HAD (ver. 18.0) and statistical significance was set at *p* < .05.

## 3 RESULTS

A total of 135 patients with stroke participated in the study (mean age: 68.9 ± 12.5 years; males, 84; females, 51; mean time since stroke: 72.5 ± 53.2 days). We excluded 28 participants because of age, inability to perform TMT or TUGT or BBS, and missing data. Finally, 107 patients with stroke were included in the analysis (mean age: 67.0 ± 11.8 years; males, 71; females, 36; mean time since stroke: 73.5 ± 55.7 days).

Classification using the TMT part B resulted in 55 participants in the ED group (prevalence: 51.4%) and 52 participants in the NED group (Table 1). The basic attributes were not significantly different between the two groups (*p *> .05; Table 1). However, time since stroke was significantly longer in the ED group than in the NED group (*p *< .05).

**TABLE 1:** Characteristics of participants.

Variables	Overall	ED group	NED group	*p* value
	(*N* = 107)	(*N* = 55)	(*N* = 52)	
Age (years)	67.0 ± 11.8	67.4 ± 13.8	66.6 ± 9.5	.746*
Gender (male/female)	71/36	37/18	33/19	.690**
BMI (kg/m^2^)	22.6 ± 3.3	22.9 ± 3.4	22.2 ± 3.2	.515*
Type of stroke (infarction/hemorrhagic)	57/50	26/29	31/21	.246**
Paretic side (right/left)	43/74	24/31	32/20	.469**
Time since stroke (day)	73.5 ± 55.7	84.8 ± 60.9	61.6 ± 47.3	.014*

*Note*: Data are expressed as mean ± standard deviation.

Abbreviations: BMI, body mass index; ED, executive dysfunction; NED, nonexecutive dysfunction. ^**^Chi‐square test, ^*^Mann–Whitney *U*‐test.

TMT part A and B were significantly slower values in the ED group than in the NED group (TMT part A: *Z* = −3.89; TMT part B: *Z* = −6.68, *p* < .001; Table 2). MMSE and FIM cognitive part score were significantly lower in the ED group than in the NED group (MMSE; *Z* = −2.58, *p* = .010, FIM cognitive part score; *Z* = −3.71, *p* < .001; Table 2).

**TABLE 2:** Results of executive function and motor function in overall participants and both groups.

Assessments	Overall	ED group	NED group	*p* value
	(*N* = 107)	(*N* = 55)	(*N* = 52)	
TMT part A (s)	77.3 ± 57.8	82.4 ± 46.2	55.5 ± 24.2	<.001*
TMT part B (s)	156.5 ± 103.8	202.4 ± 93.3	103.0 ± 67.7	<.001*
MMSE (points)	27.8 ± 2.2	27.3 ± 2.4	28.5 ± 1.8	.010*
TUGT (s)	14.25 ± 9.29	16.07 ± 9.20	16.81 ± 5.77	.008*
BRS upper‐limb	5 (1–6)	5 (1–6)	5 (3–6)	.164
BRS finger	5.5 (1–6)	5 (1–6)	5.5 (2–6)	.057
BRS lower‐limb	6 (1–6)	5 (1–6)	5 (3–6)	.452
SIAS tactile sense	3 (1–3)	3 (0–3)	3 (0–3)	.701
SIAS position sense	3 (1–3)	3 (1–3)	3 (1–3)	.635
BBS (points)	49.7 ± 5.4	46.7 ± 6.9	49.9 ± 5.6	.025*
FIM‐motor (points)	73.1 ± 15.9	69.5 ± 14.0	72.6 ± 16.0	.149
FIM‐cognitive (points)	29.5 ± 4.9	27.8 ± 5.3	31.1 ± 4.9	<.001*
FIM total (points)	102.9 ± 19.3	97.2 ± 17.6	103.8 ± 20.0	.023*

*Note*: Data are expressed as mean ± standard deviation or median (minimum–maximum).

Abbreviations: BBS, Berg balance scale; BRS, Brunnstrom recovery stage; ED, executive dysfunction; FIM, functional independence measure; MMSE, mini mental state examination; TMT, trail making test; TUGT, timed up and go test; SIAS, stroke impairment assessment set. ^*^ *p* < .05.

The TUGT, BBS, and FIM total scores were significantly slower and lower in the ED group than in the NED group (TUGT; *Z* = −2.65, *p* = .008, BBS; *Z* = −2.24, *p* = .025, FIM total score; *Z* = −2.27, *p* = .023, Table 2). However, there were no significant differences in the BRS, sensory part of SIAS, and FIM motor scores (*p* > .05).

For the results of the partial correlation analysis with age, MMSE, and FIM cognitive part score as a control factor, the TMT part B was positively correlated with TMT part A (*ρ* = 0.660, *p* < .001) and TUGT (*ρ* = 0.444, *p* < .001). The TMT part B was negatively correlated with BRS upper limb (*ρ* = −0.213, *p* = .030), finger, lower limb (*ρ* = −0.334, *p* = .001), SIAS of position sensory (*ρ* = −0.264, *p* = .007), and BBS (*ρ* = −0.331, *p* = .001).

**FIGURE 1:**
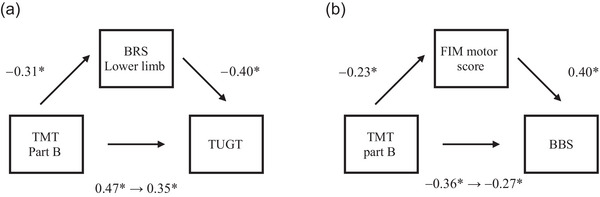
Results of the model of mediation analysis. (a) The timed up and go test (TUGT) model. The results of the mediation analysis showed that the correlation coefficient between trail making test (TMT) part B and TUGT decreased, indicating a significant Brunnstrom recovery stage (BRS) lower mediated effect. (b) The Berg balance scale (BBS) model. The results of the mediation analysis showed that the correlation coefficient between TMT part B and BBS decreased, indicating a significant functional independence measure (FIM) motor score‐mediated effect. *P < 0.05.

In the results of the mediation analysis of the TUGT model, the correlation coefficient between TUGT and TMT part B was reduced by using BRS lower limb as a mediating variable (*ρ* = 0.47–0.35). In addition, a bootstrap CI was 0.003–0.023 and to exhibit a significant positive effect (*Z* = 1.99; *p* = .047; Figure 1a). In the BBS model, the correlation coefficient between BBS and TMT part B was reduced by using FIM motor score as a mediating variable (*ρ* = −0.36 to −0.27). In addition, a bootstrap CI was −0.013 to −0.002 and showed a significant negative effect (*Z* = −2.18; *p* = .029; Figure 1b).

## 4 DISCUSSION

This study investigated the association between executive dysfunction and balance function in patients with stroke and identified mediating factors using mediation analysis. The results suggest that executive dysfunction is associated with balance function in patients with stroke, with the degree of paralysis and ADL motor function as mediators. By identifying mediating factors through this study, the causal relationship between executive dysfunction and balance can be clarified, which will help in assessment and planning intervention methods.

### 4.1 Regarding prevalence

The ED group was 51.4% and had significantly lower TMT part A, MMSE, and FIM cognitive part scores than the NED group. Our results are consistent with those of previous studies (S. Hayes et al., 2016; Sakai et al., 2023). For the prevalence, it was reported that the percentage of patients in stroke with ED was 47%, and the percentage reported for the current study was 51.4%. S. Hayes et al. (2016) used the BADS to classify whether executive dysfunction was present or absent and examined the prevalence. The resulting prevalence rate was 47%. In the current study, TMT part B was used to define and classify those with two SD or greater as having executive dysfunction in normal participants (Ashendorf et al., 2008) and found a prevalence of 51.4%, a value similar to the previous study (Hayes et al., 2016). The BADS is an assessment that is a measure of executive dysfunction (Wilson et al., 1996), and the TMT part B is also a tool that can detect executive dysfunction (Reitan & Wolfson, 1985; Tamez et al., 2011). Therefore, we assumed that the prevalence in this study was similar to that in a previous study (S. Hayes et al., 2016).

There were no significant differences in basic attributes between the two groups, but ED group had a longer duration of disease than the NED group. The results supported the previous study (Sakai et al., 2023). A previous study reported that those with executive dysfunction had less independence in daily living and longer time since stroke than those without executive dysfunction (Sakai et al., 2023). Hence, we assumed that executive dysfunction would affect time since stroke.

### 4.2 Regarding executive and cognitive function

The ED group had lower TMT part A, MMSE, and FIM cognitive part scores than the NED group. These results are supported by those of previous studies (Lipskaya‐Velikovsky et al., 2018; Sakai et al., 2023). Sakai et al. (2023) used the TMT Part B to categorize executive dysfunction by severity and examined whether there were differences between the TMT part A and FIM cognitive items in patients with stroke. Results showed that the group with executive dysfunction had slower TMT part A scores and lower FIM cognitive items. Executive function includes performing tasks, working memory, cognitive flexibility (attention), and behavioral inhibition (Skidmore et al., 2023). These abilities are detected by TMT part A (Reitan & Wolfson, 1985; Tamez et al., 2011) and FIM cognitive scores (Granger, 1986); therefore, we assumed that the ED group had lower TMT part A and FIM cognitive items than the NED group. The TMT part A and FIM cognitive scores of the ED and NED groups were clearly different, but the difference on the MMSE was only about 1.2 points (ED group: 27.3 ± 2.4 points, NED group: 28.5 ± 1.8 points). The MMSE scores of both groups were at least above 27 points, which places them in the same category of normal cognitive and level of cognitive function (Perneczky et al., 2006). We assumed that this was due to the small SD, which may have led to the significant difference between the two groups. In other words, even when general cognitive function is preserved, executive dysfunction is associated with impaired attention and social cognition.

### 4.3 Regarding executive dysfunction and balance function

The ED group had slower TUGT score and lower BBS score than the NED group. For the correlation analysis with age, MMSE, and FIM cognitive part score as a control factor, the TMT part B was correlated with TUGT, BBS, BRS, and sensory part of SIAS. These results are supported by those of previous studies (S. Hayes et al., 2016; Liu‐Ambrose et al., 2007; Sakai et al., 2023; Yu et al., 2021). Sakai et al. (2023) investigated whether TUGT differs according to the degree of executive dysfunction in patients with stroke and found that TUGT differs according to the degree of executive dysfunction. Yu et al. (2021). also reported that executive dysfunction was associated with TUGT using correlation analysis. S. Hayes et al. (2016) investigated the association between executive dysfunction and balance in patients with stroke and reported that a factor associated with executive dysfunction was balance function as measured by the BBS. Executive functions include goal planning, task implementation, flexible thinking, and working memory (Sakai et al., 2024; Skidmore et al., 2023). TUGT requires goal planning and task execution as well as other executive functions such as getting up from a chair, walking around a cone, and sitting in a chair again (Hafsteinsdottir et al., 2014). Therefore, we assumed that executive functions would be associated with TUGT. The BBS has to do the same and execute the task correctly as instructed. Therefore, we inferred that the executive function and BBS are related because, like TUGT, the executive function is required. In addition, executive function, as measured by the TMT part B, was related to the degree of paralysis and sensory function. As the severity of motor paralysis and sensory function increased, executive function also decreased (Einstad et al., 2021; S. Hayes et al., 2016). Therefore, we assumed that these were related.

### 4.4 For the mediation analysis

Two models (TUGT and BBS) were created for the mediation analysis in this study. In the TUGT model, we hypothesized, and our hypothesis is correct, that TMT part B influenced TUGT as mediated by BRS lower limb function. TUGT is a dynamic balance index with walking and balance functions (Hafsteinsdottir et al., 2014). It has been reported that the degree of paralysis is related to walking (Chan et al., 2017), and the degree of paralysis is related to executive function (Einstad et al., 2021). Therefore, we assumed that executive function affects the TUGT via the degree of paralysis.

In the BBS model, we hypothesized that TMT part B influenced BBS mediated by FIM motor score, and this result was also correct. Previous studies have reported that balance and ADL function are associated (E. H. Kim & Cho, 2023; Shao et al., 2024), and executive function is involved in both balance and ADL function (Ghaffari et al., 2021; Kim et al., 2014; Sakai et al., 2023). Low executive function limits freedom in daily living and decreases the frequency of physical activity (Janssen et al., 2014). It was hypothesized that an increase in activity level would increase the frequency of physical activity and contribute to an improvement in balance ability. Therefore, the results of this model showed a causal relationship. By identifying mediating factors through this study, the causal relationship between executive dysfunction and balance can be clarified, which will aid in the assessment and planning of intervention methods.

This study has several limitations. First, an assessment of executive function was only conducted on the TMT part B. Therefore, it is necessary to assess various aspects in the future. The second is the failure to ask about the educational background. Educational background has been found to be a factor associated with executive function (Perpiñà Martí et al., 2023). Therefore, it is necessary to ask about it as a covariate in future studies. Third, the small number of factors assessed in this study limited the factors used in the mediation analysis. Therefore, a wide range of assessments and multifaceted analyses are needed in the future. Finally, mediation analysis can only analyze unidirectional causality. Therefore, path analysis or covariance analysis should be selected when analyzing bidirectional associations.

In conclusion, executive dysfunction and balance function were associated, and the degree of paralysis and ADL motor function associated with them in patients with stroke.

## AUTHOR CONTRIBUTIONS

**Katsuya Sakai**: Conceptualization; methodology; software; validation; formal analysis; supervision; visualization; project administration; writing—review and editing; writing—original draft. **Yuichiro Hosoi**: Methodology; validation; writing—review and editing. **Yusuke Harada**: Validation; investigation; data curation; writing—review and editing. **Yuichi Kato**: Writing—review and editing; investigation; data curation; validation.

## CONFLICT OF INTEREST STATEMENT

The authors declare no conflicts of interest.

### PEER REVIEW

The peer review history for this article is available at https://publons.com/publon/10.1002/brb3.3542.

## Data Availability

The data that support the findings of this study are available from the corresponding author upon reasonable request.
